# Linear Spatial–Numeric Associations Aid Memory for Single Numbers

**DOI:** 10.3389/fpsyg.2019.00146

**Published:** 2019-02-04

**Authors:** John Opfer, Dan Kim, Christopher J. Young, Francesca Marciani

**Affiliations:** ^1^Department of Psychology, The Ohio State University, Columbus, OH, United States; ^2^University of Chicago Consortium, Chicago, IL, United States; ^3^University of Alabama at Birmingham, Birmingham, AL, United States

**Keywords:** numerical estimation, memory development, numerical cognition, spatial–numerical association, memory, counting, cardinality knowledge

## Abstract

Memory for numbers improves with age. One source of this improvement may be learning linear spatial–numeric associations, but previous evidence for this hypothesis likely confounded memory span with quality of numerical magnitude representations and failed to distinguish spatial–numeric mappings from other numeric abilities, such as counting or number word-cardinality mapping. To obviate the influence of memory span on numerical memory, we examined 39 3- to 5-year-olds’ ability to recall one spontaneously produced number (1–20) after a delay, and the relation between numeric recall (controlling for non-numeric recall) and quality of mapping between symbolic and non-symbolic quantities using number-line estimation, give-a-number estimation, and counting tasks. Consistent with previous reports, mapping of numerals to space, to discrete quantities, and to numbers in memory displayed a logarithmic-to-linear shift. Also, linearity of spatial–numeric mapping correlated strongly with multiple measures of numeric recall (percent correct and percent absolute error), even when controlling for age and non-numeric memory. Results suggest that linear spatial–numeric mappings may aid memory for number over and above children’s other numeric skills.

## Introduction

Both in school and everyday life, children are presented with a potentially dazzling succession of numbers to remember. Some numbers must be remembered exactly, such as phone numbers and the answers to arithmetic problems. Others only need to be remembered approximately, such as the number of children in one’s class, the amount of money in one’s piggy bank, or the temperature forecast for tomorrow’s weather. When confronted with a series of numbers in either type of situation—e.g., a digit span task (Dempster, 1981) or a vignette (Brainerd and Gordon, 1994)—young children recall numbers much less accurately than older children and adults. In this paper, we examine whether developmental changes in numerical representation accounts for individual differences in memory for numbers. Specifically, we test how children’s memory for numbers relates to their memory for non-numeric information (e.g., color) and to their knowledge of numeric magnitude, indexed by their ability to map a number to a spatial location on a number line, to map a discrete number of objects to a number word, and to count.

### Fuzzy Trace Theory

The fuzzy trace theory (FTT) depicts information as being stored in memory using one of two representational formats, a short-term verbatim or “surface form” representation and a long-term gist or “fuzzy trace” representation (Reyna et al., 2009). Within this account, when numerical information is learned (e.g., Farmer Brown owns many animals. He has 5 sheep, 11 cows, and 3 dogs.), numbers can be encoded precisely using a verbatim representation (e.g., 5 sheep, 11 cows, and 3 dogs), including the specific format in which they were presented (e.g., numerals, dots, etc.). Verbatim memories preserve exact surface forms of numerical inputs for a short period of time, but lack relative concepts or relations between numbers (e.g., *the most, the least, more*, and *less*). However, the meaning of numbers is encoded as a gist representation (e.g., Farmer Brown owns more cows than dogs.). Gist memories do not contain formatting information of numerical inputs, but preserve a sense of approximate magnitude or relative amount (e.g., *about six*, *less*, *more*, *a lot*, etc.) for a longer period of time (Brainerd and Gordon, 1994).

An attractive feature of FTT is that it helps to explain what changes in memory development. That is, for young children, memory for numerical information in the verbatim representation is superior to that of the gist representation, but this advantage attenuates with age. Thus, by adulthood, there is greater reliance on longer lasting, but imprecise, gist representations of numerical magnitude (Brainerd and Gordon, 1994). Empirical support for this account was shown by dissociation between performance on relative comparisons of quantity in gist tests (e.g., “Which of Farmer Brown’s animals are the most, cows or dogs?”) and exact identification of quantity in verbatim memory tests (e.g., “How many cows does Farmer Brown own, 11 or 3?”). Between preschool and second grade, gist recall increased with age and ultimately surpassed verbatim recall, which did not change with age.

While FTT provides accurate predictions for general improvement via an age-related switch to gist memory for numbers, it is not clear whether it provides a sufficient mechanism for how features of the stimulus, such as the magnitude of a to-be-remembered number, will influence the likelihood of its recall. One idea had been that “physical distinctiveness” helps to distinguish items in the verbatim representation and thereby improve memory (Brainerd and Reyna, 1993; Brainerd and Gordon, 1994, p. 166). Under this view, the physical distinctiveness of some items in verbatim tests is greater than that of others. For example, when choosing whether 12 vs. 10 cows had been studied, the physical difference between the two two-digit numbers is less than the physical difference between 12 vs. 3 cows. Consistent with this idea, greater numerical distance improves young children’s memory more than it does older children’s memory.

### Representational Change Account

A somewhat different depiction of the distance effect—and how symbolic numeric information is stored in memory—comes from findings on development of numerical magnitude representations (for review, see Opfer and Siegler, 2012). As we will see, this account also depicts representations of numeric magnitude as being “fuzzy” and approximate. However, unlike FTT, the internal magnitudes associated with numbers are also depicted as changing with development, such that the distinctiveness of the memory trace changes more for large than small numbers.

A coherent picture of how numerical magnitude representations change with age and experience is provided by previous research on numerical estimation. In early development, young preschoolers attach no cardinal value to numerical symbols, and they do not yet map numeric symbols like number words and Arabic numerals (even approximately) to non-symbolic numeric quantities. For example, 2- and 3-year-olds who count flawlessly from 1 to 10 have no idea that 6 > 4, nor do preschoolers of these ages know how many objects to give an adult who asks for 4 or more (Le Corre et al., 2006; though see Lee and Sarnecka, 2010). Later, as non-mapping children gain experience associating numeric symbols with real-world quantities (such as sets of objects or number of sounds), they initially map numeric symbols to a noisy, logarithmically compressed mental number line which represents and stores the magnitudes of non-symbolic quantities (such as objects and tones) in memory. During this period, preschoolers know approximately how many objects to give an adult who asks for 1–20 objects and approximately where on the number-line 1–20 fall, but their estimates in each case increase logarithmically with the number to be estimated (Berteletti et al., 2010; Opfer et al., 2010; Thompson and Siegler, 2010; Kim and Opfer, 2017).

Development from a logarithmic to linear representation of numeric value occurs iteratively. Over a period that typically lasts 1–3 years for a given numerical range (0–10, 0–20, 0–100, or 0–1,000), children’s mapping of symbolic numbers to non-symbolic quantities changes from a logarithmically compressed form to a linear form, where subjective and objective numerical values increase in a 1:1 fashion (Siegler and Opfer, 2003; Siegler and Booth, 2004; Opfer and Thompson, 2008; Thompson and Opfer, 2008, 2010; Berteletti et al., 2010; Opfer et al., 2010). Use of linear numerical-magnitude representations occurs earliest for the numerals that are most frequent in the environment (i.e., the smallest whole numbers) and is extended to increasingly large numbers with experience (Thompson and Opfer, 2010). Although some alternative models (e.g., Gallistel and Gelman, 1992; Ebersbach et al., 2008; Cantlon et al., 2009; Moeller et al., 2009; Barth and Paladino, 2011; Cohen and Sarnecka, 2014) have argued that the mapping of symbolic numbers to magnitudes does not show an abrupt transition from a precisely logarithmic to a precisely linear representation (but see Opfer et al., 2011, 2016; Young and Opfer, 2011; Kim and Opfer, 2017; Qin et al., 2017), all models capture a similar phenomenon—young children estimate the magnitudes of small numbers as differing more than the magnitudes of large numbers, whereas older children and adults estimate the magnitudes in a more closely 1:1 fashion.

Associations between linear numerical-magnitude representations and numeric memory were recently explored by Thompson and Siegler (2010), who presented children with numbers in a vignette and asked them to recall the numbers after a brief distracter task (naming four cartoon characters). For example, children were given a story, “Colleen washes the dishes at a restaurant. This month, she washed *N*_1_ forks, *N*_2_ cups, and *N*_3_ plates.” After a distractor task, they were asked how many forks, cups, and plates Colleen washed respectively. Several observations from Thompson and Siegler (2010) suggested that linear numerical-magnitude representations aided memory for numerical information. First, linearity and accuracy on approximate magnitude tasks (number-line estimation and number categorization) were highly correlated with number memory, whereas accuracy on non-approximate magnitude tasks (counting and number identification) was not. Thus, a third variable (such as overall numeric proficiency) was unlikely to be a source of the positive correlation between numerical estimation and memory. Second, memory accuracy measured using the percent absolute error (PAE) deteriorated with the magnitude of the number given, especially for children with a logarithmic representation of number (Thompson and Siegler, 2010, Experiment 3). This finding is important because if numeric symbols are mapped with a constant noisiness to a logarithmically scaled mental number line, then signal overlap increases dramatically with numerical value, thereby leading to significant interference from adjacent values as the target number increases. Interference from highly similar exemplars is a well-known source of errors in recall (Schacter et al., 1998), yet it would not be predicted if children’s memory for numbers depended solely on their memory span. Finally, preschoolers’ difficulty recalling large numbers could not be explained by large numbers simply being unfamiliar to them. When preschoolers were tested to see how high they could count, Thompson and Siegler (2010) observed no correlation between the largest number counted and memory accuracy.

### The Current Study

In this study, we investigated a potential source of concern about evidence supporting the representational change account. That is, individual and developmental differences also exist in memory span; the number of digits that can be accurately recalled at age 2 years is about 2, at age 5 about 4, at age 10 about 5, and among adults 7 ± 2 (Dempster, 1981). Thus, given that memory span and linear numerical-magnitude representations (in the 1–20 range) develop simultaneously, a spurious correlation between linearity of numerical estimation and span-based numerical memory may have been observed by Thompson and Siegler (2010) because children were asked to remember *multiple* items that exceeded their memory span.

This concern seems particularly justified by two previous findings. First, a correlation exists between working memory span and linear numerical-magnitude representations (e.g., Geary et al., 2007). Second, the sum of items and distractors in Thompson and Siegler (2010) study would have been at the edge of many children’s memory span, leading many children to fail to recall numeric information if memory span were a contributor to numerical memory.

To address these concerns, the current study tested 3- to -5-year-olds’ memory *for a single number*, thereby obviating any potential contribution of individual differences in memory span to numerical memory, and children’s memory *for a single color*, in order to control for non-numeric memory ability. As in Thompson and Siegler (2010; Experiment 2), we examined (1) preschoolers’ recall of numbers 1–20 because preschoolers vary in whether they represent these numbers as increasing linearly (Berteletti et al., 2010; Thompson and Siegler, 2010), (2) the degree to which preschoolers’ estimates of positions of numbers on number lines increased linearly with actual numeric value, and (3) preschooler’ counting from 1 to 20. Additionally, we examined (4) children’s performance on a “give-a-number” task (Wynn, 1990) because estimates on this task have been reported to show a logarithmic-to-linear shift (Opfer et al., 2010) and to provide a more robust test of number understanding than counting accuracy (Wynn, 1990; Le Corre et al., 2006; Sarnecka and Carey, 2008).

Our predictions regarding quantitative performance were derived from research on development of numerical abilities. Generally, we predicted development across all three tasks would ultimately involve accurate and linear mappings between symbols and quantities, but the developmental paths to this would be more similar for number-line and give-a-number estimation than counting. This is because accurately translating from numerical magnitudes to symbolic numbers can be accomplished procedurally (by counting), without knowing the 1:1 mapping of symbols and quantities more generally (Briars and Siegler, 1984; Wynn, 1990). In contrast, accurately translating from a symbolic number to a magnitude requires this knowledge, and it develops slowly from non-mapping (i.e., random translation between symbols and magnitudes) to noisy non-linear mapping to precise linear mapping (i.e., systematic translation between symbols and magnitudes) (Siegler and Opfer, 2003; Opfer et al., 2010; Wagner and Johnson, 2011). Thus, among children who map symbols to quantity in the number-line estimation and/or the give-a-number task, we expected a significant increase in linearity with age along with an increase in accuracy. In contrast, among non-mappers, we expected no age-related improvements in linearity or accuracy.

Our predictions regarding memory performance were derived from the representational change account. Specifically, if linearity of numeric magnitude representations influences the likelihood that numbers are recalled accurately, then preschoolers with the most linear mappings on our estimation tasks would likely recall numbers the most accurately as well. Further, if representations of numeric magnitude develop from a logarithmic (or similarly compressed) mapping to a linear mapping over preschool, an interaction between age and magnitude on memory accuracy would be expected. That is, young and old preschoolers would have nearly equally accurate memories for small numbers, whereas young preschoolers would have significantly less accurate memories for large numbers than older preschoolers. If so, linearity of representation would likely mediate the relations between age and number memory that increases with age.

## Materials and Methods

### Participants

Thirty-nine preschoolers (54% female) were recruited from six child-care centers in the Columbus metro area. Preschoolers were aged 3 years (*n* = 13, *M* = 3.63), 4 years (*n* = 14, *M* = 4.45), and 5 years (*n* = 12, *M* = 5.35). Mean age of all children was 4.45 years. Only children whose parents or guardians had provided written consent and who verbally agreed to take part in the research participated in the study. All task materials and experimental procedures described below were approved by the Institutional Review Board at The Ohio State University.

### Tasks

For all tasks, preschoolers were presented with eight numbers in randomized order. We presented the same numbers used in Berteletti et al. (2010): 2, 4, 6, 7, 13, 15, 16, and 18 to each child on each task.

#### Number-Line Estimation

The number-line estimation task was adapted from Siegler and Opfer (2003). Preschoolers were presented with a sheet of paper on each trial of the task. Centered on each sheet was a 25-cm line, flanked by two vertical hatch marks. The value “0” was written below the vertical hatch mark representing the left end of the line, and the value “20” was written below the mark representing the right end of the line. Above the middle of the line was one of the 8 task numerals, centered within a circle. The experimenter told the child, “Today, we’re going to play a game with number lines. What I’m going to ask you to do is show me where on the number line some numbers are. When you decide where the number goes, I want you to make a mark through the number line like this,” and demonstrated marking the line. All numbers were read aloud, but preschoolers were not corrected on their responses nor told that the halfway position along the line is where “10” should go.

#### Give-a-Number Estimation

The give-a-number estimation task was adopted from Wynn (1990). Preschoolers were presented with a pile of 20 blue poker chips and told that the experimenter would ask them for a number of chips. The child’s task was to place what he or she believed to be the correct number of chips before the experimenter, and the experimenter confirmed the child’s response by asking, “And how many is that?”

#### Counting Task

In the counting task, preschoolers were presented a 72-cm black poster board strip. Attached to each strip were a number of white poker chips that were presented to each child so that the first chip was at the left end of the strip, with each successive chip centered 4 cm to the right of the previous one. Each child was told, “You have to find out how many chips there are on this card.” Children were neither encouraged nor discouraged to count, so that they would use their own strategies. Thus, although it was possible for children to estimate the number of chips, most children of all ages counted chips aloud from left to right.

#### Number/Color Recall Task

The numerical recall task was intertwined with the counting task. A recall trial immediately followed each counting trial. After explaining the counting task to the child, the experimenter indicated a second experimenter at a separate location within the same room. The experimenter then instructed the child that he or she was to tell the second experimenter a “password” and how many chips there were on the card. The “password,” designed to prevent children from rehearsing the number prior to recall, was the color of construction paper presented by the experimenter. Upon reaching the second experimenter, the child was asked the “password” (color), and then how many chips were on the card (number). Thus, by testing recall of numbers and colors that children had generated themselves, we could be certain that the items to be remembered were familiar to children and had been encoded.

### Design and Procedure

Testing was administrated in two sessions. In a first session, children played one of two games based on Siegler and Ramani (2009). In each game, 22 colored squares of identical size were ordered consecutively on a board. The first square was labeled “Start,” and the last square was labeled “Finish.” Squares between the first and last were consecutively numbered from 1 to 20. The sole difference between the games was the arrangement of numbers. In one game, numbered squares were placed in a horizontal line across the board, arranged left-to-right. In the other game, numbered squares were arranged in a circle, with numbers increasing in value in a clockwise direction. In the games, children were asked to move their token from “Start” to “Finish” and read the numbers on the squares as they moved. The games were included to test effects of the spatial arrangement of numbers on children’s numerical understanding. Unlike Siegler and Ramani (2009), however, there were no main (or interactive) effects of game type on number and memory tasks for the second session, presumably due to the much shorter time allotted for game play in the present study.

In a second session, experimenters revisited schools within 4 days to administer the battery of tasks described above (i.e., number-line, give-a-number, counting, and recall tasks). Order of presentation was counterbalanced, with the exception that the recall task necessarily followed the counting task. There were no carry-over effects of particular task order (*ps* > 0.05). Children were tested individually during one 25-min session occurring in a quiet room in their school.

## Results

Our results are divided into two major sections. In the first section (“Description of Task Performance”), we describe age-related changes in preschoolers’ number-line and give-a-number estimation, counting, and recall. In the next section (“Logarithmic Compression in Numerical Tasks as Predictors of Memory Performance”), we examine relations among quantitative performance and recall. One 4-year-old who completed number-line and give-a-number tasks but did not complete counting and recall tasks was excluded from analyses that involved the two incomplete tasks.

### Description of Task Performance

For our quantitative tasks, we examined accuracy and linearity of the mapping between numeric symbols and quantities. Accuracy was measured using the mean percent absolute error (MPAE) scores for a child. Within each trial, PAE for number-line estimation was calculated using the formula, (|Number Presented-Number Estimated|/20)^∗^100, for give-a-number estimation using (|Chips Requested-Chips Given|/20)^∗^100, and for counting using (|Chips Shown-Number Counted|/20)^∗^100. The MPAE was then computed by obtaining the across-trial mean of the PAEs.

To calculate linearity, children’s responses of the three tasks were fitted by a mixed log-linear model (MLLM) (Anobile et al., 2012; Opfer et al., 2016), formalized as follows:

$$y=a\left(\lambda \frac{U}{\ln(U)}\ln(x)+(1-\lambda )x\right).$$

In the MLLM, *a* denotes a scaling parameter, *U* the upper bound, *x* a given magnitude, and *y* a child’s estimate. It also includes a logarithmic component (λ) that measures a degree of logarithmic compression in responses and is constrained to be between 0 and 1. If estimates are perfectly logarithmic, λ equals 1, whereas λ is 0 for perfectly correct and linear estimates. The MLLM was fitted to each child individually and to median responses collapsed across children by age group.

#### Number-Line Estimation

**FIGURE 1:**
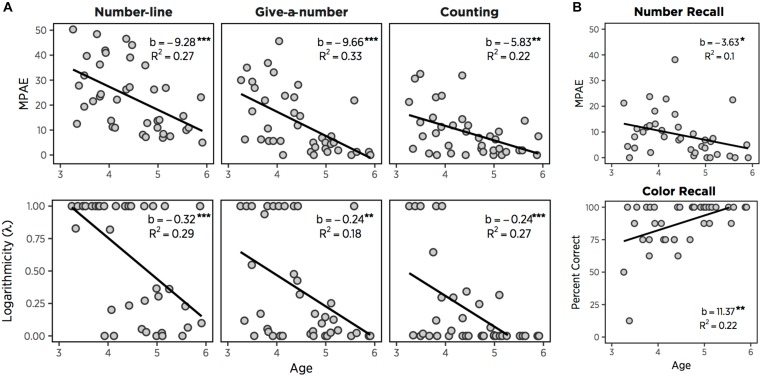
MPAE and logarithmic components (λ) as a function of age in three numerical tasks **(A)**. MPAE of number recall and the percent correct of color recall **(B)**. *^∗^p* < 0.05, *^∗∗^p* < 0.01, *^∗∗∗^p* < 0.001.

As expected, estimation accuracy (MPAE) improved significantly with age, *b* = −9.281, *t*(37) = −3.67, *p* < 0.001. Also, linearity measured with logarithmic components (λ) improved with age, *b* = −0.316, *t*(37) = −3.90, *p* < 0.001 (Figure 1A). Age also explained 26.6% of variance in accuracy, *F*(1,37) = 13.44, *p* < 0.001, and 29.1% of variance in linearity, *F*(1,37) = 15.19, *p* < 0.001. The average MPAE for all children was 23.16 (*SD* = 13.47), and the averaged value of logarithmic components was 0.61 (*SD* = 0.44) (Table 1). The MPAE was 32.19 (*SD* = 11.75) for 3-year-olds, 23.68 (*SD* = 13.59) for 4-year-olds, and 12.76 (*SD* = 6.56) for 5-year-olds. The average logarithmic component (λ) for 3-year-olds was 0.91 (*SD* = 0.28), 0.61 (*SD* = 0.44) for 4-year-olds, and 0.29 (*SD* = 0.36) for 5-year-olds. These results are broadly consistent with the “logarithmic-to-linear shift” in number-line estimation (Siegler et al., 2009).

**Table 1:** Mean performance (and SDs) by tasks for each age group.

		Age			
Task	Measure	3-year-old	4-year-old	5-year-old	Total
Number-line	MPAE	32.19 (11.75)	23.68 (13.59)	12.76 (6.56)	23.16 (13.47)
	Log component	0.91 (0.28)	0.61 (0.44)	0.29 (0.36)	0.61 (0.44)
Give-a-number	MPAE	20.38 (12.26)	13.35 (13.19)	4.32 (5.99)	12.92 (12.64)
	Log component	0.53 (0.47)	0.40 (0.43)	0.13 (0.28)	0.36 (0.42)
Counting	MPAE	14.62 (10.92)	8.13 (8.54)	3.85 (4.39)	9.00 (9.38)
	Log component	0.47 (0.47)	0.09 (0.12)	0.03 (0.09)	0.20 (0.35)
Color recall	Accuracy (%)	79.81 (25.79)	86.54 (13.94)	96.88 (5.65)	87.5 (18.38)
Number recall	Accuracy (%)	48.08 (26.44)	55.77 (23.73)	76.04 (24.11)	59.54 (26.86)
	MPAE	10.78 (7.26)	10.94 (10.19)	4.48 (6.39)	8.84 (8.48)

*MPAE, mean percent absolute error.*

Previous work explained age-related changes in accuracy of preschoolers’ number-line estimates as coming from a logarithmic to linear shift in representations of numerical magnitude (Berteletti et al., 2010; Thompson and Siegler, 2010). Consistent with this idea, the accuracy measure (MPAE) significantly correlated with logarithmic components in number-line estimation *r*(36) = 0.64, *p* < 0.001 (Table 2). The association remained strongly even after controlling for age, *r*(35) = 0.50, *p* < 0.01. Besides, we found that linearity of estimates improved with age. Median estimates of 3-year-olds increased more logarithmically with actual value, whereas estimates of 4- and 5-year-olds increased more linearly with actual value compared to younger children (3s: λ = 1, 4s: λ = 0.34, 5s: λ = 0.14). Thus, at the group level, all three age groups mapped numeric magnitudes at least approximately to the number line, with logarithmic compression decreasing with age.

**Table 2:** Correlations among numeric memory measures and predictor variables.

	1	2	3	4	5	6	7	8	9
1. Number recall accuracy (%)									
2. Number recall MPAE	−0.75^∗∗∗^								
3. Age	0.48^∗∗^	−0.32^∗^							
4. Color recall accuracy (%)	0.21	−0.29	0.47^∗∗^						
5. NL MPAE	−0.48^∗∗^	0.42^∗∗^	−0.53^∗∗^	−0.23					
6. GAN MPAE	−0.44^∗∗^	0.43^∗∗^	−0.59^∗∗∗^	−0.57^∗∗∗^	0.68^∗∗∗^				
7. Counting MPAE	−0.13	0.35^∗^	−0.47^∗∗^	−0.53^∗∗^	0.49^∗∗^	0.73^∗∗∗^			
8. NL log component	−0.46^∗∗^	0.42^∗∗^	−0.54^∗∗^	−0.19	0.64^∗∗∗^	0.41^∗^	0.31		
9. GAN log component	−0.38^∗^	0.41^∗^	−0.42^∗∗^	−0.55^∗∗∗^	0.55^∗∗∗^	0.89^∗∗∗^	0.66^∗∗∗^	0.29	
10. Counting log component	−0.18	0.11	−0.52^∗∗^	−0.55^∗∗∗^	0.53^∗∗^	0.76^∗∗∗^	0.76^∗∗∗^	0.19	0.71^∗∗∗^

*MPAE, mean percent absolute error; NL, number-line task; GAN, give-a-number task. Asterisks indicate levels of significance (^∗^p < 0.05, ^∗∗^p < 0.01, ^∗∗∗^p < 0.001).*

To test whether individual children approximately mapped the magnitude of symbolic numbers to their number-line estimates as well, we next evaluated whether each child’s estimates increased with the numbers presented to them. To do this, we categorized children into two categories, mapping and non-mapping groups, by using a goodness of fit measure (*R*^2^) of the MLLM. Children whose estimates did not increase progressively with given magnitudes and were not explained by a MLLM at all (i.e., *R*^2^ = 0) were considered as non-mappers, whereas children whose estimates were accounted for by a MLLM (i.e., *R*^2^ > 0 regardless of the statistical significance of *R*^2^) were categorized as mappers (but see Sella et al., 2017, for mapper categorization using a simple linear or log function). The non-mappers (*n* = 17), constituted 43.6% of all preschoolers (69.2% of 3-year-olds, 42.9% of 4-year-olds, and 16.7% of 5-year-olds). These results indicate a significant difficulty among the majority of children, particularly 3- and 4-year-olds, in mapping symbolic numbers even approximately to their corresponding positions on the number-line.

**FIGURE 2:**
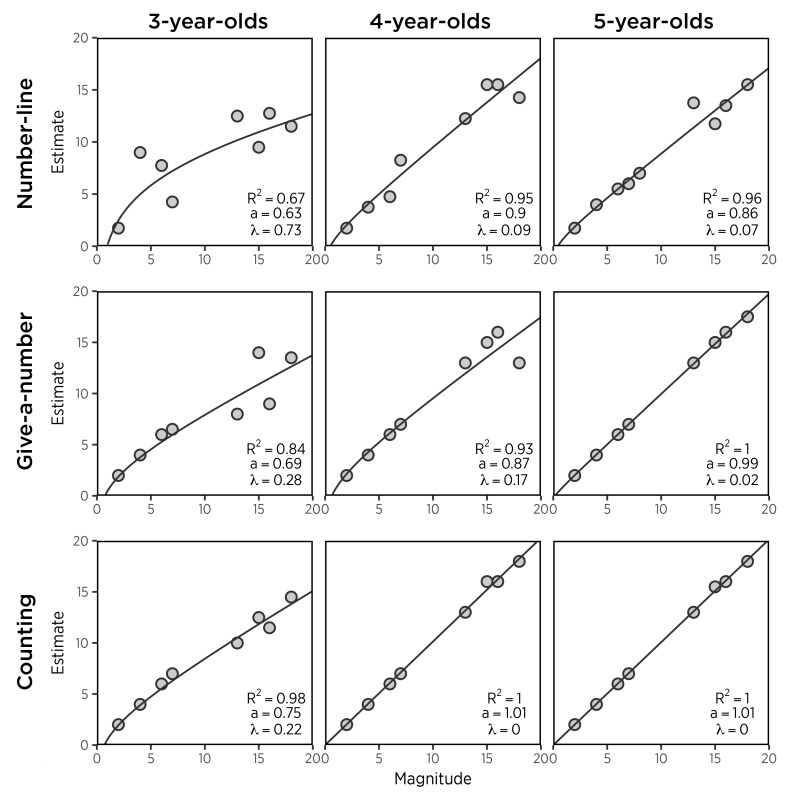
Median responses on quantitative tasks in mapping children by age group.

Among preschoolers who did not show this difficulty in mapping numbers to the number line (*n* = 22), however, there was stronger support for a logarithmic-to-linear shift. First, we observed significant age-related improvements in linearity, *b* = −0.319, *t*(20) = −3.88 *p* < 0.001, and in accuracy, *b* = −6.417, *t*(20) = −2.57, *p* < 0.05. Age explained 42.9% of variance in linearity, *F*(1,20) = 15.05, *p* < 0.001, and 24.7% of variance in accuracy, *F*(1,20) = 6.59, *p* < 0.05. Consistent with the individual level analyses, median estimates of mappers were more linear with age. As shown in Figure 2, median estimates by 3-year-olds were logarithmically compressed (λ = 0.73), whereas 4- and 5-year-olds produced linear median estimates (4s: λ = 0.09, 5s: 0.07). In contrast, non-mapping children (*n* = 17) showed no effect of age on either linearity or accuracy (*p*s > 0.05). These results suggest that while mapping children show a developmental progression in the linearity of the representation used on the number line estimation task as well as better accuracy, non-mapping children seem to have had such a poor understanding of the mapping of numeric magnitudes to linear distance that neither their accuracy nor their linearity improved with age^1^.

#### Give-a-Number Estimation

Give-a-number estimation accuracy also improved significantly with age, *b* = −9.662, *t*(37) = −4.25, *p* < 0.001, as did linearity, *b* = −0.240, *t*(37) = −2.86, *p* < 0.01 (Figure 1A). Age explained 32.8% of variance in accuracy, *F*(1,37) = 18.03, *p* < 0.001, and 18.1% of variance in linearity, *F*(1,37) = 8.16, *p* < 0.01. The average MPAE for all children was 12.92 (*SD* = 12.64), and average logarithmic component was 0.36 (*SD* = 0.42) (Table 1). The MPAE was 20.38 (*SD* = 12.26) for 3-year-olds, 13.35 (*SD* = 13.19) for 4-year-olds, and 4.32 (*SD* = 5.99) for 5-year-olds. The average logarithmic component for 3-year-olds was 0.53 (*SD* = 0.47), 0.40 (*SD* = 0.43) for 4-year-olds, and 0.13 (*SD* = 0.28) for 5-year-olds.

Previous work had explained age-related changes in accuracy of preschoolers’ give-a-number estimates as coming from a logarithmic to linear shift in representations of numerical magnitude (Opfer et al., 2010). Consistent with this idea, the correlation between accuracy and linearity measures was considerable [*r*(36) = 0.89, *p* < 0.001; Table 2], even when age was controlled [*r*(35) = 0.88, *p* < 0.001]. We also observed the median number of chips given by 3-year-olds increased logarithmically with the number of chips requested (λ = 0.68), whereas chips given by 4- and 5-year-olds increased linearly with the number requested (4s: λ = 0.19, 5s: 0.02). Thus, at the group level, all three age groups appeared to map numeric magnitudes at least approximately to the number of chips they provided, with superiority of the linear to the logarithmic functions increasing with age.

We next tested for an approximate mapping between number and magnitude on the give-a-number test at the individual level. As done on the number-line estimation test, we regressed the number of chips requested by an experimenter against the number of chips given by each child; the proportion of non-mapping children, whose estimates were not explainable by a MLLM, was calculated. Children were categorized into either non-mappers or mappers using the goodness of fit criterion (i.e., *R*^2^). In total, only 10.3% of all preschoolers showed non-significant correlation between the chips given and the number requested (three 3-year-olds and one 4-year-old). These results indicate a better understanding of the task than observed for number-line estimation, though many younger children still failed to map symbolic number to the number of chips even in an approximate fashion.

To test the hypothesized learning sequence, we next evaluated age as a predictor of both linearity and accuracy measures. Mapping children (*n* = 35) showed a marginally significant improvement in linearity, *b* = −0.17, *t*(33) = −2.02, *p* = 0.052, and accuracy, *b* = −7.08, *t*(33) = −3.71, *p* < 0.001. Age explained 11% of variance in linearity, *F*(1,33) = 4.07, *p* = 0.052, and 29.4% of variance in accuracy, *F*(1,33) = 13.77, *p* < 0.001. Median estimates increased more logarithmically with actual value in 3- and 4-year-old mappers (3s: λ = 0.28, 4s: λ = 0.17), compared to 5-year-old mapping children whose median estimates were almost perfectly linear (λ = 0.02) (Figure 2). Thus, like the results from number-line estimation, results suggest that preschoolers who approximately map number to magnitude show a log-to-linear progression in the representation used.

#### Counting Task

Counting accuracy improved significantly with age, *b* = −5.83, *t*(36) = −3.20, *p* < 0.01, as did linearity, *b* = −0.24, *t*(36) = −3.63, *p* < 0.001 (Figure 1A). Age also explained a significant percentage of variance in accuracy, 22.1%, *F*(1,36) = 10.22, *p* < 0.01, and in linearity, 26.8%, *F*(1,36) = 13.25, *p* < 0.001. The average MPAE for all children was 9.00 (*SD* = 9.38), and average logarithmic component was 0.20 (*SD* = 0.35) (Table 1). The MPAE was 14.62 (*SD* = 10.92) for 3-year-olds, 8.13 (*SD* = 8.54) for 4-year-olds, and 3.85 (*SD* = 4.39) for 5-year-olds. The average logarithmic component for 3-year-olds was 0.47 (*SD* = 0.47), 0.09 (*SD* = 0.12) for 4-year-olds, and 0.03 (*SD* = 0.09) for 5-year-olds.

Previous work had explained age-related changes in accuracy of preschoolers’ counting as coming from procedural knowledge rather than representational change (Briars and Siegler, 1984; Wynn, 1990). Somewhat surprisingly, however, we found significant relations between accuracy and linearity in counting no matter whether age effects were partialled out [*r*(36) = 0.76, *p* < 0.001 for the zero-order correlation; *r*(35) = 0.69, *p* < 0.001 for the partial correlation]. In addition, median counts of 3-year-olds increased more logarithmically with the number of chips presented (λ = 0.38), whereas median counts of 4- and 5-year-olds increased perfectly linearly with chips presented (4s: λ = 0, 5s: λ = 0). Thus, at the group level, all three age groups appeared to map numeric magnitudes at least approximately to the number of chips they provided, with superiority of the linear to the logarithmic functions increasing with age.

Because, as we have seen, that analyses of group data are not always consistent with analyses of individual performance, we next tested for an approximate mapping between number and magnitude at the individual level, as we did on the two estimation tests. As in number-line and give-a-number tasks, children were divided into two groups, mapping and non-mapping groups, based on fitting of a MLLM to their responses. Only one 3-year-old child was found to have no relation between the chips given and the number requested. Without the one non-mapper, the age effects in linearity and accuracy still stayed significant at both individual and group levels. For example, after taking out the non-mapper, median responses of 3-year-olds still increased more logarithmically with actual given magnitudes (λ = 0.22), whereas 4- and 5-year-olds’ median responses were completely linear (4s: λ = 0, 5s: λ = 0), suggesting log-to-linear developmental shifts in counting (Figure 2). Taken together, these results show that in general most 3- to 5-year-old preschoolers were capable of approximately mapping the number of chips to their counts and that there were developmental progresses in their mapping.

Is the developmental path of counting different from that of number-line and give-a-number estimation that requires deep understanding of number-to-magnitude mappings? Our results from accuracy and linearity in counting above suggest that even though number-to-magnitude mapping is not necessary in counting, it follows log-to-linear shifts as in other estimation tasks (Figure 1). However, counting appeared to be more accurate and linear than the other tasks. As shown in Figure 2, median responses of mapping children increased more linearly from the age three and reached perfect linearity earlier in counting than number-line and give-a-number estimation. Consistent with median responses, individual children performed better in counting than the other tasks (MPAE: *M*_NL_ = 23.16, *M*_GAN_ = 12.92, *M*_count_ = 9.0; λ: *M*_NL_ = 0.61, *M*_GAN_ = 0.40, *M*_count_ = 0.20). Whereas counting accuracy correlated with accuracy of estimation tasks, *rs* = 0.49-0.73, *p* < 0.01, linearity in counting showed no association with number-line estimation, but only with give-a-number estimation, *r*(36) = 0.71, *p* < 0.001 (Table 2). Together, even if counting shares the developmental trajectory with estimation, counting improves faster than estimation.

#### Number/Color Recall Task

We next examined age-related improvements in the percentage of colors recalled (Figure 1B). Color recall also showed a strong effect of age, *b* = 11.37, *t*(36) = 3.58, *p* < 0.01, with age explaining 22.9% of variance, *F*(1,36) = 10.09, *p* < 0.01. Overall, the percentage of colors recalled accurately improved with age, (3s, *M* = 79.81%, *SD* = 25.79%; 4s, *M* = 86.54%, *SD* = 13.94%; 5s, *M* = 96.88%, *SD* = 5.65%) (Table 1). To compare color vs. number memories, we computed the percentage of numbers correctly recalled (e.g., correct if children recall 5 and incorrect if they recalled 6 after counting 5). When two types of recall accuracy were compared, color recall was superior to number recall, *t*(37) = 5.89, *p* < 0.001, presumably due to its greater temporal proximity.

To calculate MPAE for the number recall task, we took the average of the Percentage Absolute Error (PAE), or (|Number Counted–Number Remembered)|/20^∗^100, across all trials for children. Thus, if a child (correctly or incorrectly) said there were 12 chips on a card and then recalled there being 13 chips, PAE would be 5%.

To examine development of numeric recall, we carried out a regression between age and the percentage of numbers recalled perfectly, as well as between age and MPAE in recalling the numbers they initially counted. As expected, the recall of the exact numbers improved with age, *b* = 17.07, *t*(36) = 3.29, *p* < 0.01, and age explained 23.1% of variance, *F*(1,36) = 10.8, *p* < 0.01. The mean percentage of exact number recall trials was 48.08% (*SD* = 26.44%) for 3-year-olds, 55.77% (*SD* = 23.73%) for 4-year-olds, and 76.04% (*SD* = 24.11%) for 5-year-olds (Table 1). More importantly, younger children recalled numbers that were more deviated from correct numbers, whereas older children retrieved numbers more accurately (Figure 1B). Recall MPAE improved with age, *b* = −3.63, *t*(36) = −2.05, *p* < 0.05, and age explained 10.1% of variance, *F*(1,36) = 4.21, *p* < 0.05. The average MPAE for all children was 8.84 (*SD* = 8.48). The mean of individuals’ MPAE was 10.78 (*SD* = 7.26) for 3-year-olds, 10.94 (*SD* = 10.19) for 4-year-olds, and 4.48 (*SD* = 6.39) for 5-year-olds. As expected, then, we observed age-related increases in the percentage of numbers recalled perfectly and decreases in MPAE of numeric recall.

**FIGURE 3:**
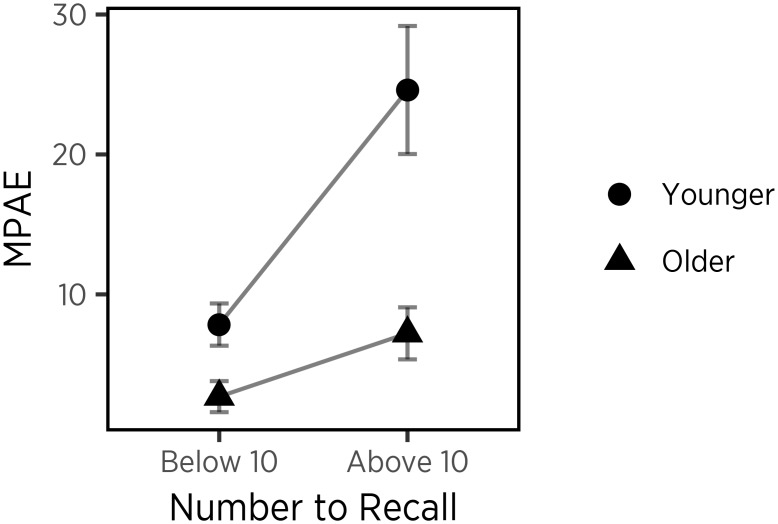
Relation between the magnitude of the number to be recalled and error in recall performance, for younger (circles) and older (triangles) children. Error bars represent standard errors.

To test the representational change account of recall more directly, we examined memory MPAE for an interaction of age and numeric magnitude. This interaction is predicted uniquely by the idea that the subjective magnitudes of numbers change with age. According to the representational change account, children should show good recall for small numbers regardless of representation. However, for large numbers, only children who possess a linear representation would be able to distinguish them from one another, whereas children with a logarithmic representation would show more erroneous memory for large numbers due to greater overlaps in representation. To test this, we divided number recalls into two categories based on the number of digits that a stimulus contained (i.e., numbers below 10 vs. numbers above 9) to control for visual features shared by single-digit and two-digit numbers. Children were also divided into two groups relative to the median split of ages for children in the study (4.43 years old). As predicted, a mixed ANOVA showed a main effect of numeric magnitude on recall accuracy, *F*(1,34) = 17.79, *p* < 0.001, a main effect of age group on recall accuracy, *F*(1,34) = 18.90, *p* < 0.001. More importantly, an interaction effect of numeric magnitude and age group on recall accuracy, *F*(1,34) = 5.59, *p* < 0.05 (see Figure 3). *Post hoc* comparisons using Bonferroni’s adjustment revealed that younger children generated significantly greater errors for larger numbers (*M* = 24.60, *SD* = 3.45) than for smaller numbers (*M* = 8.54, *SD* = 1.39), *p* < 0.001, whereas errors of smaller numbers (*M* = 2.69, *SD* = 1.31) did not differ from those of larger numbers (*M* = 7.22, *SD* = 3.26) in older children, *p* = 0.19. Taken together, the results suggest that younger children with a logarithmic representation produced greater errors for larger numbers that have more overlaps with other numbers in representation, supporting the representational change account.

### Logarithmic Compression in Numerical Tasks as Predictors of Memory Performance

Might improvement in memory accuracy—like improvement in accuracy and linearity of numerical estimates, give-a-number estimates, and counting—be related to increasingly linear representations of numerical value? Correlations among tasks show that it might be the case. As shown in Table 2, numeric memory measured with percent correct and MPAE was strongly associated with accuracy and linearity of number-line and give-a-number estimates, but weakly correlated with those of counting. To test this more closely, we conducted multiple regression analyses on the mean percent deviation between recalled and correct numbers (MPAE) in the recall task. In the analyses, children’s age, percent correct responses in color recall, and average value of numbers to recall were entered in a regression model to control for the influences of the variables. The mean of to-be-recalled numbers varied across children because the stimuli were generated by individual children in the counting task. In addition to the three predictors, MPAEs from three numerical tasks were included in a regression model in order to examine the unique contributions of the numeric tasks to the MPAE in number recall simultaneously.

The model accounted for a significant amount of variance in recall MPAE (64%), *F*(6,31) = 9.14, *p* < 0.001. The errors in children’s number recall were explained by the mean values of numbers that children produced themselves to recall later [*b* = 3.02, β = 0.77, *t*(31) = 5.97, *p* < 0.001]. Children who produced larger magnitudes on average in counting tended to produce more erroneous responses in number recall. Interestingly, the accuracy of number-line estimation was another significant predictor for the number recall accuracy [*b* = 0.22, β = 0.35, *t*(31) = 2.20, *p* < 0.04]. Whereas give-a-number MPAE was marginally predictive of recall performance [*b* = 0.30, β = 0.41, *t*(31) = 1.95, *p* = 0.06], MPAE in counting did not explain errors in number recall [*b* = −0.04, β = −0.04, *t*(31) = −0.27, *p* = 0.79]. Interestingly, whereas age was a significant predictor for number memory in a simple regression (*b* = −3.63, *p* < 0.05), the age effects were not evident in the multiple regression, where the age variable was tested with five other predictors [*b* = −1.79, β = −0.16, *t*(31) = −1.11, *p* = 0.28]. Neither was the color recall accuracy (percent correct) a significant predictor for the number recall performance [*b* = −8.65, β = −0.19, *t*(31) = −1.31, *p* = 0.20].

Using the same analyses, we next tested whether linearity of some tasks better predicted numerical memory than that of other tasks. Individuals’ MPAEs from the three numeric tasks were replaced with their respective logarithmic components (λ) as predictors. More than 66% of variance in number recall accuracy was addressed by the six predictors (age, percent of correct responses in color recall, average value of numbers to recall, and λs for number-line estimation, give-a-number estimation, and counting), *F*(6,31) = 10.44, *p* < 0.001. Again, age and accuracy in color memory did not predict number recall significantly [*b* = −1.82, β = −0.16, *t*(31) = −1.11, *p* = 0.28 for age, *b* = −6.39, β = −0.14, *t*(31) = −1.05, *p* = 0.30 for color memory]. On the other hand, the mean number that children produced to remember has a considerable contribution to MPAE in number recall [*b* = 2.98, β = 0.76, *t*(31) = 5.38, *p* < 0.001]. More importantly, degrees of linearity in both number-line and give-a-number estimation accounted for performance in number recall [*b* = 7.48, β = 0.39, *t*(31) = 3.02, *p* < 0.01 for number-line linearity, *b* = 9.00, β = 0.44, *t*(31) = 2.82, *p* < 0.01 for give-a-number linearity]. The logarithmic component in counting did not associate with number recall in a significant way [*b* = 1.25, β = 0.05, *t*(31) = 0.27, *p* = 0.79].

Next, extending the multiple regressions, we conducted mixed-effects modeling on trial-to-trial PAEs of number recall with varying intercepts for participants to investigate relations between number memory and numerical tasks. The mixed-effects model allows for examining average (fixed) effects of numerical tasks on number memory across children while also accounting for individual differences among children. In the analysis, fixed effects included children’s age, color recall accuracy, number to recall, and MPAEs from number-line estimation, give-a-number estimation, and counting. The *p*-values for fixed effects were computed using Satterthwaite’s approximation method (Satterthwaite, 1941) to define denominator degrees of freedom for the *t*-test. Intercepts were treated as random at the participant level to control for inter-individual variability. When the effects of the six variables were averaged over all children, the number that children produced to recall was the only significant fixed effect [*b* = 1.52, β = 0.52, *t*(31) = 10.58, *p* < 0.001], implying that PAE in number recall increased with the magnitude of numbers to recall. Neither age nor accuracy measures of three numerical tasks showed significant effects on accuracy in number recall.

Another linear mixed-effects analysis was conducted on PAEs for every trial in number recall. The model was identical to the one described above except that the MPAEs from the numeric tasks were replaced with their respective logarithmic components (λ). Again, fixed effects of numbers to recall was significant [*b* = 1.51, β = 0.52, *t*(31) = 10.47, *p* < 0.001], indicating that number recall accuracy varied depending on the magnitude of to-be-recalled numbers. Using the linearity instead of accuracy, the model showed significant effects of logarithmic components of number-line estimation and give-a-number tasks [*b* = 5.94, β = 0.14, *t*(31) = 2.26, *p* < 0.05 for number-line estimation, *b* = 10.59, β = 0.23, *t*(31) = 3.13, *p* < 0.01 for give-a-number]. The results suggest that the more logarithmic in the two numerical tasks, the fuzzier number recall performance, and that the linearity indices reliably predict number memory. The linearity measure from counting was not significant [*b* = −4.54, β = −0.08, *t*(31) = −1.04, *p* = 0.30]. Again, age and accuracy of color recall did not contribute to number recall.

## Discussion

Previous work has indicated that development of linear representations of numerical magnitudes profoundly expands children’s quantitative thinking (Opfer and Siegler, 2012). It improves children’s ability to estimate the positions of numbers on number lines (Siegler and Opfer, 2003; Siegler and Booth, 2004; Opfer and Siegler, 2007), to estimate the measurements of continuous quantities (Thompson and Siegler, 2010) and the quantity of discrete objects (Opfer et al., 2010), to categorize numbers according to size (Laski and Siegler, 2007; Opfer and Thompson, 2008), and to estimate and learn the answers to arithmetic problems (Booth and Siegler, 2008; Kim and Opfer, 2017; Qin et al., 2017). Recent work has also indicated that the logarithmic-to-linear shift is associated with improved memory for numbers (Thompson and Siegler, 2010; Thompson and Opfer, 2016).

In this paper, we took a critical look at the representational change theory of development of numerical recall. We were particularly interested in whether it could account for changes in ability to recall single numbers that children themselves produced. This issue is important because previous work could not rule out the influence of memory span on numerical memory. Further, by examining numbers that children themselves produced, we could eliminate the possibility that preschoolers with non-linear numerical-magnitude representations were simply poor at remembering unfamiliar numbers. Thus, we sought to provide a robust test of the theory.

Consistent with the representational change account, we found preschoolers’ recall of a single number to be closely tied to the linearity of their mapping between numeric symbols and quantities. Indeed, this connection to preschoolers’ numerical recall was even beyond what would be expected based solely on their age or memory for other items, such as self-generated color words. Further, consistent with the hypothesis that young children are unable to correctly recall large numbers due to increasing semantic similarity among large numeric magnitudes, we found that young preschoolers’ memory for small numbers was nearly equivalent to that of older preschoolers’ memory, whereas older preschoolers recalled large numbers much more accurately than younger preschoolers. An intriguing question for future research is the extent to which the semantic similarity of numbers co-varies with other forms of similarity (e.g., phonological or visual form; Cohen, 2009) and which type of similarity best predicts numeric recall.

In addition, our results showed that children’s performance in number recall was predicted by accuracy and, more reliably, by linearity in number-to-quantity mapping tasks—i.e., number-line estimation and give-a-number tasks—as well as by the magnitude of numbers to recall. Surprisingly, the effects of age and memory for non-numerical items on number recall were not evident when all predictors were considered simultaneously. The findings remained consistent no matter whether the effects were examined with single-level or multi-level analyses. Together, the findings provide strong evidence for the representational change account.

Theoretically, the effect of numerical magnitude on recall accuracy is important because it suggests a new way to integrate findings regarding development of memory and numerical cognition. That is, both areas of research strongly suggest that long-duration representations of numerical magnitude are “fuzzy” and approximate (Gallistel and Gelman, 1992; Brainerd and Reyna, 1993; Dehaene and Changeux, 1993; Brainerd and Gordon, 1994). However, unlike the findings integrated by the FTT, findings on development of numerical estimation suggest that the internal magnitudes associated with numbers change over development from a logarithmic to a linear association (Siegler and Opfer, 2003; Opfer and Siegler, 2007; Siegler, 2016), with the result that the distinctiveness of the representation of numeric magnitudes is initially larger for small numbers than large ones. The implication of this view for numeric recall comes from the general finding that the probability of recall is positively related to the distinctiveness of the representation in memory (Greene and Crowder, 1984), with the apparently correct prediction that recall accuracy would be initially greater for small numbers than large numbers and that this difference would decline with age. Previous work has demonstrated that adults produce non-linear estimates of very large numbers (e.g., a million) as young children do for small numbers (Landy et al., 2013, 2017). Given that compressive number-to-magnitude mapping is not limited to children, whether adults’ memories for numbers are also subject to magnitudes is an interesting question for future research.

Beyond demonstrating that linear numerical-magnitude representations are associated with improved memory for numbers, the present results also help to explain the positive relation between linear numeric magnitude representations and arithmetic proficiency (Booth and Siegler, 2008; Kim and Opfer, 2017; Qin et al., 2017). That is, if developing linear numerical-magnitude representations improves memory for single numbers (e.g., four chips) as well as multiple numbers presented in vignettes (e.g., four cows, six cows), it is highly likely that it also improves memory for numbers in other contexts, such as memorizing arithmetic facts (e.g., 4 cows + 6 cows = 10 cows). In this way, the present results suggest a plausible explanation for the observed association between numerical estimation and mathematics course grades (Booth and Siegler, 2008; Halberda et al., 2008), and it suggests that numerical memory may moderate this link. Although this account is admittedly speculative, we believe it is an important issue for future research.

## Author Contributions

JO designed the experiments, analyzed the data, and co-wrote the manuscript. DK and CY analyzed data and contributed to writing of the manuscript. FM collected the data.

## Conflict of Interest Statement

The authors declare that the research was conducted in the absence of any commercial or financial relationships that could be construed as a potential conflict of interest.

## Footnotes

1 One may argue that the MLLM is not the best model for young children’s estimates, and their number-line performance may be better explained by a variety of cyclic power models (Barth and Paladino, 2011; Slusser et al., 2013; Cohen and Sarnecka, 2014; Cohen and Quinlan, 2018). To test this, we also fitted the mixed cyclic-power models (MCPM1 and MCPM2; see Kim and Opfer, 2017, for details), the mixtures of diverse cyclic-power models that have been proposed in literature (Opfer et al., 2016; Kim and Opfer, 2017; Qin et al., 2017). When the MCPMs were compared with the MLLM using corrected Akaike information criterion (AICc) and Bayesian information criterion (BIC), the MLLM was the best-fitting model for median estimates of all three age groups and all individual children’s estimates regardless of which model-selection criterion was used. Furthermore, the weight parameters of sub-models in MCPMs (i.e., *w*_1_, *w*_2_, and *w*_3_) that are thought to capture developmental changes presented no systematic patterns that might reflect age-related improvement.
